# The NeoRoo mobile app: Initial design and prototyping of an Android-based digital health tool to support Kangaroo Mother Care in low/middle-income countries (LMICs)

**DOI:** 10.1371/journal.pdig.0000216

**Published:** 2023-10-25

**Authors:** Sherri Lynn Bucher, Allison Young, Madison Dolan, Geetha Priya Padmanaban, Khushboo Chandnani, Saptarshi Purkayastha

**Affiliations:** 1 Department of Pediatrics, Division of Neonatal-Perinatal Medicine, Indiana University School of Medicine, Indianapolis, Indiana, United States of America; 2 Department of Community and Global Health, Richard M. Fairbanks School of Public Health, Indiana University–Indianapolis, Indianapolis, Indiana, United States of America; 3 Scholarly Concentration in Public Health Certificate Program, Indiana University School of Medicine and Richard M. Fairbanks School of Public Health, Indiana University–Indianapolis, Indianapolis, Indiana, United States of America; 4 Department of Human Centered Computing, Human-Computer Interaction, Luddy School of Informatics, Computing, and Engineering, Indiana University–Indianapolis, Indianapolis, Indiana, United States of America; 5 Department of BioHealth Informatics, Data Science and Health Informatics, Luddy School of Informatics, Computing, and Engineering, Indiana University–Indianapolis, Indianapolis, Indiana, United States of America; Iran University of Medical Sciences, IRAN (ISLAMIC REPUBLIC OF)

## Abstract

Premature birth and neonatal mortality are significant global health challenges, with 15 million premature births annually and an estimated 2.5 million neonatal deaths. Approximately 90% of preterm births occur in low/middle income countries, particularly within the global regions of sub-Saharan Africa and South Asia. Neonatal hypothermia is a common and significant cause of morbidity and mortality among premature and low birth weight infants, particularly in low/middle-income countries where rates of premature delivery are high, and access to health workers, medical commodities, and other resources is limited. Kangaroo Mother Care/Skin-to-Skin care has been shown to significantly reduce the incidence of neonatal hypothermia and improve survival rates among premature infants, but there are significant barriers to its implementation, especially in low/middle-income countries (LMICs). The paper proposes the use of a multidisciplinary approach to develop an integrated mHealth solution to overcome the barriers and challenges to the implementation of Kangaroo Mother Care/Skin-to-skin care (KMC/STS) in LMICs. The innovation is an integrated mHealth platform that features a wearable biomedical device (NeoWarm) and an Android-based mobile application (NeoRoo) with customized user interfaces that are targeted specifically to parents/family stakeholders and healthcare providers, respectively. This publication describes the iterative, human-centered design and participatory development of a high-fidelity prototype of the NeoRoo mobile application. The aim of this study was to design and develop an initial (“A”) version of the Android-based NeoRoo mobile app specifically to support the use case of KMC/STS in health facilities in Kenya. Key functions and features are highlighted. The proposed solution leverages the promise of digital health to overcome identified barriers and challenges to the implementation of KMC/STS in LMICs and aims to equip parents and healthcare providers of prematurely born infants with the tools and resources needed to improve the care provided to premature and low birthweight babies. It is hoped that, when implemented and scaled as part of a thoughtful, strategic, cross-disciplinary approach to reduction of global rates of neonatal mortality, NeoRoo will prove to be a useful tool within the toolkit of parents, health workers, and program implementors.

## Author summary

We aim to leverage the promise of digital health to overcome barriers and challenges to neonatal care, particularly thermal care, vital signs monitoring, and Kangaroo Mother Care/Skin-to-skin care (KMC/STS), in low/middle-income countries. Our multidisciplinary team has developed an integrated mHealth platform, composed of a wearable biomedical device called NeoWarm, which is a self-warming swaddling pouch plus baby carrier with sensors for automated vital signs monitoring, combined with a mobile app called NeoRoo. NeoRoo is a permission-based app with two user interfaces, customized for parents/family stakeholders and healthcare providers of premature and low birthweight babies. The app provides information, support, and resources designed to equip and empower adult stakeholders of prematurely born babies to more effectively adopt and implement KMC/STS, an evidence-based neonatal intervention that has been shown to significantly reduce the incidence of neonatal hypothermia and improve survival among premature infants. Using a human-centered and participatory design approach, we performed iterative development efforts for the open-source NeoRoo app from August 2020 to February 2021. This resulted in a high-fidelity prototype with features and functions for automated vital signs monitoring, communication between parents and health workers, shared goal-setting and tracking for key KMC/STS metrics, and educational resources.

## Introduction

High rates of premature birth (defined as delivery prior to 37 weeks gestation), and neonatal mortality (NMR; defined as the death of liveborn infants from the day of delivery to 28 days postnatal), are urgent global challenges. Annually, there are an estimated 2.5 million neonatal deaths. Fifteen million babies are born prematurely every year, and complications from prematurity are the leading cause of death among children under the age of five years. [1] Despite significant global progress over the past 30 years in reducing deaths due to prematurity [2], there remains a disproportionate burden of mortality due to complications of prematurity within low/middle-income countries (LMICs), particularly within Sub-Saharan Africa and Asia. [3,4]

Neonatal hypothermia–the inability of infants to maintain their body temperature above 36.5°C (97.7°F)—is an extremely widespread complication among prematurely born and low birthweight (small) babies [5], and a significant cause of morbidity (illness) and contributing factor to mortality (death) among newborns. [6,7] Globally, it is estimated that around 17 million newborns in LMICs each year suffer from hypothermia. [8] In the sub-Saharan Africa setting, rates of neonatal hypothermia as high as 60–85% have been reported. [9,10] Within high-income settings, prematurely born infants are typically managed within incubators, by highly skilled healthcare providers (HCPs) with years of specialized training in neonatal care. Within LMIC settings, however, lower availability of incubators and radiant warmers [11,12], high patient volumes (especially within newborn units), [13] and a paucity of physicians and nurses [14–16] contribute to unacceptably high rates of neonatal hypothermia and hypothermia-related newborn mortality.

Kangaroo Mother Care/Skin-to-skin care (KMC/STS) is an evidence-based method of newborn care involving skin-to-skin contact between an adult caregiver and infant, which has been shown to significantly reduce the incidence of neonatal hypothermia within LMICs [17], and greatly improve rates of survival among premature infants. [18–24]Challenges and barriers to adopting and implementing KMC/STS have been identified to exist for all the key adult stakeholders within a premature baby’s environment, including among parents. [25–27] and healthcare providers. [28,29]

Affordable digital health solutions that can be adopted at the health facility level might hold promise to improve care for premature babies, including overcoming barriers to adoption of KMC/STS, and strengthening implementation. Over the past decade, mobile phone ownership, including of smart phones, and access to cellular networks has surged. [30–32] Acceptance of, and access to, a wide variety of digital health interventions using mobile devices (mHealth) has skyrocketed among all stakeholders, including healthcare providers. [33–37] Implementation of mHealth and digital tools in LMICs have been demonstrated to have numerous benefits, including improving access to care for patients, [38,39] increasing knowledge retention and competencies among community health workers, [40,41] and improving training outcomes, confidence/satisfaction, and reporting of key indicators among health providers. [35,42–44]

### Landscape for newborn care

Within many LMICs, including Kenya, midwives and nurses provide most of the care for mothers and babies; unfortunately, there is a profound global shortage of nurses, particularly those specialized in neonatal care. [13,45] As a result, nurse:newborn patient ratios in some health facilities in Kenya can range from 1 nurse:7–15 newborns, [16] or even exceed 1:25+. [15] By contrast, in resource-rich settings, neonatal intensive care unit (NICU) nurse:patient ratios are typically 1:1 to 1:3. [46] A NICU nurse:patient ratio of 1:4 in the United States has been demonstrated to result in missed nursing care. [47] The nurse:newborn patient ratios observed in Kenya and other LMIC health systems are significantly higher than that recommended within guidelines for staffing ratios from organizations such as the National Association of Neonatal Nurses. [48] Evidence-based neonatal care recommendations from international partners such as WHO indicate that some key vital signs should, ideally, be measured in hospitalized newborns around every 4 hours, around the clock. However, at one large referral hospital in Kenya, it was demonstrated that only 63% of premature babies had *at least 1* temperature taken and only 53% had *at least one* respiratory and heart rate measurement recorded. [49]

### Description of prior mobile app development

From 2016 to present, our Indianapolis-based team, in conjunction with partners at Moi University, Kenya and a variety of international stakeholders, have designed and developed five Android apps, purposively integrated with the District Health Information System 2 (DHIS2; [50]) for newborn care education, training, data collection, and clinical decision support, as digital tools to augment the American Academy of Pediatrics’ *Helping Babies Survive* programs. These mobile *Helping Babies Survive* powered by DHIS2 apps (mHBS/DHIS2) include digital tools to support the *Helping Babies Breathe* (HBB) and *Essential Care for Every Baby* (ECEB) programs. [51]

Three mHBS/DHIS2 apps, to support training and education for neonatal resuscitation (HBB), were recently utilized in a randomized control trial among 274 maternity nurses from 20 healthcare facilities in Kenya and Nigeria, and were found to be feasible, acceptable, and exceptionally robust. [52,53] An award-winning clinical decision support app developed by our team, ECEB/mHBS, provides comprehensive support for healthcare providers in LMICs to deliver evidence-based essential newborn care interventions from birth through 24 hours after delivery. [54,55] We have also built a digital health tool, “NeoLinkID,” that is designed to support birth registration and secure portability of mother-baby health records in LMICs. [56]

NeoRoo mobile applications expand the mHBS/DHIS2 suite to equip healthcare workers and parents of premature/small babies with digital tools to support an integrated continuum of support for KMC/STS. With NeoRoo, we seek to alleviate barriers to KMC/STS, a proven method of reducing mortality in premature infants, and empower parental caregivers, families, and healthcare workers with education and tools to deliver high-quality, evidence-based care.

We propose to leverage the promise of digital health to overcome known barriers, challenges, and gaps in the care of premature/small babies and to the implementation of facility-based KMC/STS in LMICs. Our multidisciplinary team has developed a suite of innovations to provide automated thermal support and vital signs monitoring for premature/low birthweight infants while they are engaged in KMC/STS with adult caregivers. Our innovation is an integrated mHealth platform that features a wearable, sensor-enabled, self-warming, biomedical device (swaddling baby carrier) with automated vital signs monitoring for KMC/STS adult-baby dyads (“NeoWarm”; [57,58]) and connected Android-based mobile applications which are customized, respectively, for parents/family stakeholders and healthcare providers (“NeoRoo”).

Within the scope of this paper, we describe the human-centered design and iterative development of high-fidelity prototypes of an initial (“A”) version of the NeoRoo mobile application and highlight key features and functions that have resulted from these efforts. This paper describes iterative app design and high-fidelity prototype development activities from August 2020 –February 2021.

## Materials and methods

### Ethics approval

This study was determined to be “Non-Human Subjects Research” (#15181) by the Indiana University Institutional Review Board based on the following criteria: (1) neither the biomedical device (NeoWarm) nor the app (NeoRoo) are currently being studied according to the FDA’s definition; (2) this is not a safety/efficacy study; (3) this was development of a mobile app prototype that would not be tested in the clinical setting within the current study; (4) the study was conducted purely for product design and development purposes. Because this study was determined by the IU IRB to be “Non-Human Subjects Research,” formal consent was not obtained.

### Study setting

The use case for the NeoRoo app is to support adult caregivers (parents/families and healthcare providers) of premature or small babies within KMC/STS wards in Kenyan health facilities to provide evidence-based neonatal care more effectively.

#### Information, communication, and technology (ICT) ecosystem in Kenya

Kenya, located in East Africa, is ideally suited for digital health and mHealth solution studies. It has a very robust ICT ecosystem, with governance and policy development lead by the Ministry of Information, Communications, and Technology and the ICT Authority Kenya. [59] Kenya boasts a 70% internet penetration rate [60] and has been dubbed “The Silicon Savannah,” [61] because it is a global leader in mHealth and digital innovation. [60] Of particular interest, one of the stated priorities of The Silicon Savannah is to support rapid acquisition toward 2030 Sustainable Development Goals, including reduction of neonatal mortality. Thus, the overall goals of the NeoRoo initiative, reduction of morbidity and mortality among prematurely born and low birthweight babies, also overlaps with the stated purposes of national partners in Kenya.

#### IU-Kenya Global Health Equity Partnership

The NeoRoo app design and development efforts are performed within the context of a long-term research and educational partnership between Indiana University School of Medicine, in Indianapolis, Indiana, and Moi University College of Health Sciences Eldoret, Kenya. From 1989 to the present, the Indiana University School of Medicine and Moi University College of Health Sciences have participated in the IU-Kenya Partnership. [62–66] The IU-Kenya Partnership also supports the Academic Model Providing Access to Healthcare (AMPATH), which, with USAID support, serves a population of 13 million persons at over 300 Ministry of Health facilities across a 17-county catchment area. Thus, for purposes of iterative participatory design and development of mHealth innovations, the NeoRoo team has access to a wide variety of stakeholders and potential end-users across all levels of the health system and health service delivery continuum within Kenya.

### Study design

#### Overall methodological approach

**Fig 1:**
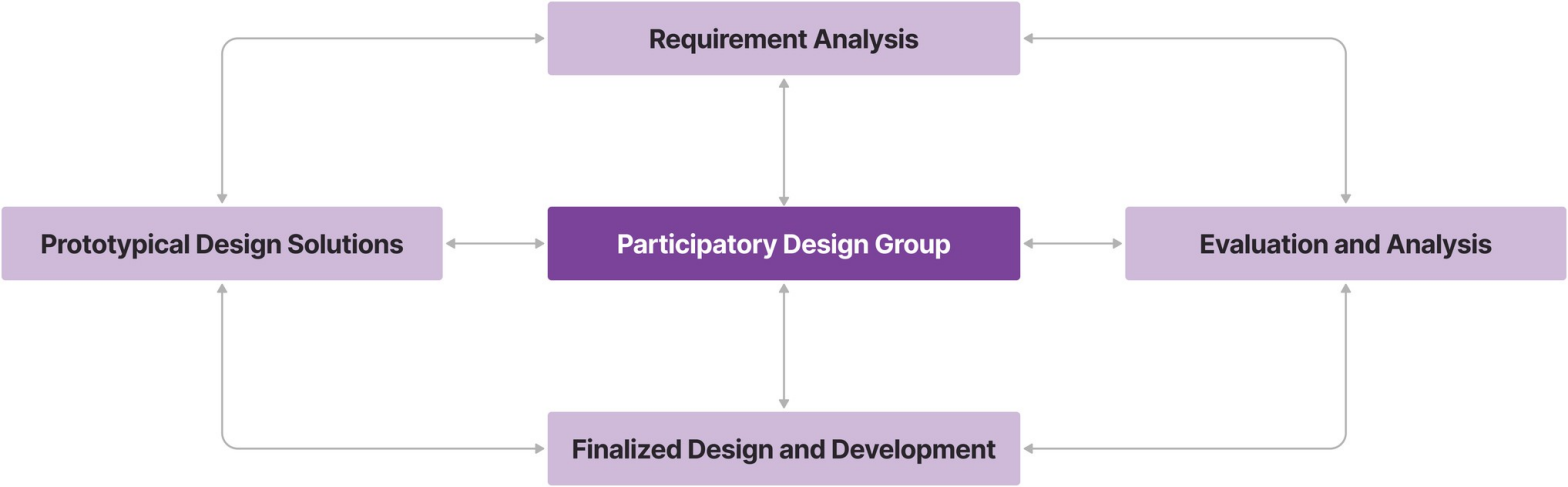
NeoRoo mHealth design and development efforts are continuous, iterative, and anchored by human-centered participatory design efforts. We utilize a variety of evaluation and analysis methods, as further described in the text.

To develop mHealth and biomedical device innovations, our collaborative team utilizes a combined human/User-centered [67] and participatory design approach, [68–70] and incorporates principles of Design Thinking. [71] These activities, in turn, are firmly nested within a comprehensive, multi-faceted, integrated effort to improve the adoption, uptake, and delivery of proven, evidence-based interventions for premature/small babies. We employ iterative, agile development techniques. [72] Across our iterative design and development efforts, we utilize best practices in regards to user interface (UI) and user experience (UX) design [see also, Table 3 in Horsky et al.,2012 [73]], and, in close collaboration with our international partners, employ successive cycles of participatory design, underpinned by heuristic evaluation, to ensure that the digital tools and platforms we build are feasible, acceptable, and effective, within the settings where they are deployed (Fig 1).

Data security and privacy protections are paramount; the mHealth apps that we build are GDPR compliant. We build our mHealth solutions to maximize the potential for wide adoption and sustainable scale-up; as such, we align our efforts with those currently on-going among key global partners across various landscapes and ecosystems, such as the World Health Organization’s *Every Newborn Action Plan* (ENAP) metrics group and WHO Quality, Equity, Dignity Network, for the capture of indicators regarding KMC/STS, quality-of-care, and newborn mortality and morbidity. [74] Our mHealth solutions are built to align with the 9 Principles for Digital Development, endorsed by a wide variety of partners within the international development and global health space. [75] Our mobile app development efforts are incubated within the open-source LibreHealth collaborative community, according to FLOSS (Free/Libre and Open-Source Software) development principles. We partner hand-in-glove with LMIC partners, to ensure that we “*Design for the User*,” “*Understand the Existing Ecosystem*,” and “*Be Collaborative*” (Principles for Digital Development 1, 2, 9).

#### Description of the connected biomedical device

A complete description of the biomedical device is provided in Bluhm et al., 2020, [57] and within the US 1039063 B2 patent application, *Infant thermoregulation and monitoring support system*. [76] Briefly, the biomedical device is a carrier (worn by an adult) and integrated self-warming, sensor-enabled swaddling pouch with 4 flaps (worn by the premature baby). When an adult caregiver and infant engage in KMC/STS, the flaps open to allow for skin-to-skin contact. When the dyad is not engaged in KMC/STS, if the adult caregiver wishes to lay the baby on a cot or other surface (“stand-alone” mode), the flaps of the swaddling pouch snap closed to prevent thermal loss in the newborn via conduction and convection. Regardless of the mode of use (i.e., whether during “KMC/STS” or “stand-alone” mode), sensors in the device provide continuous vital signs monitoring of 4 key physiological parameters in the newborn (body temperature, respiration, heart rate, and blood oxygen saturation). There are also visual and audio alarms integrated within the biomedical device, to alert caregivers, for example, when a baby’s body temperature drops too low. Bluetooth low energy technology is used to transmit information from the biomedical device to Android devices that are loaded with the NeoRoo mobile app. [57]

### Data collection and analysis

#### Composition of the app development team

The NeoRoo design and development team, comprised of Faculty, students, and technical advisors, was highly interdisciplinary, representing a broad range of collective expertise across continents (North America; sub-Saharan Africa; India) and scientific and technological domains, including: health informatics, human-computer interaction, UI/UX development, clinical care (pediatrics; neonatology; midwifery), biomedical engineering, public health, maternal-newborn-child (MNCH) health, monitoring and evaluation, and implementation science. The app design and development activities reported in this paper include: (1) user requirements analysis; (2) development of information architecture (whiteboarding); (3) user interface and user experience design (wireframes); (4) iterative development of high-fidelity prototypes (2 cycles).

#### User requirements analysis

We utilized 3 primary methods to ascertain key pain points within the existing cascade for neonatal thermal care and KMC/STS in LMICs and generate an initial list of desired user requirements (Box 1). First, a sub-set of co-authors (SLB; AY; MD) conducted a thorough literature review of both published (PubMed) and gray literature (Google Scholar) to obtain background information, on behalf of the larger team, regarding the existing landscape for KMC/STS, thermal care/neonatal hypothermia, and existing digital health applications for newborn care. Particular focus was directed toward obtaining and collating published literature regarding barriers, challenges, gaps, facilitators, and enablers for KMC/STS in LMICs, with specific emphasis on global regions with the highest neonatal mortality rates, namely, sub-Saharan Africa (especially Kenya) and Asia.

**Box 1. NeoRoo user design requirements**

- Off-line functionality, combined with automated syncing when app is on-line
- Locally adaptable & customizable
- Ability to integrate with OpenMRS and DHIS2
- Automated, “at a glance” vital signs monitoring
- Augmented decision support
- “First, do no harm:” align app content, functions, and features with existing international, national, and local guidelines and recommendations for facility-based KMC/STS and care of premature/small babies
- Scaffold improved information exchange and shared goal-setting between parents and HCPs
- Create space for potential task-sharing (between various cadres of HCPs; between HCPs and family stakeholders)
- Reduce cognitive burden
- Another useful tool in the toolkit of care for some premature or small babies, as determined by local end-users and stakeholders – not an attempt at a “silver bullet” solution
- One permission-based app, 2 targeted user interfaces

Second, our team drew upon our collective experience in global health and health informatics implementation, and prior mHealth development efforts, to inform the user requirements analysis. Advisors also included US-based neonatologists, Kenyan nurses, nurse-midwives, physicians, and public health experts. As previously described, NeoRoo is part of a suite of mobile newborn care apps that, from 2016 to the present, have collectively undergone extensive contextual analysis, human-centered design, heuristic evaluation, and randomized control testing. [51–56,77] Thus, lessons learned from this prior work also directly informed design and development efforts for the NeoRoo mobile application, an approach that adheres to the “*Reuse and improve*” and *“Be Data Driven*” Principles for Digital Development. [75]

The final method used to perform user requirements analysis was to assess results from a separate feasibility, acceptability, and user-design feedback study that was conducted independently for the NeoWarm biomedical device (IU IRB #1602698245) Those findings indirectly informed the NeoRoo team’s app development efforts by providing information confirming some of the challenges and pain points specifically faced by Kenyan adult stakeholders of premature babies. For that qualitative study (manuscript in preparation), parents, healthcare providers, and community stakeholders of small/premature babies reported that, similar to findings described in other published studies from LMIC settings, the barriers, gaps, and challenges to KMC/STS and care for premature/small babies specific to the Kenyan setting span multiple implementation domains, including sociocultural expectations, perceived gender roles, [78] lack of resources, [79] and worries about safety. [28,80,81]

#### Development of NeoRoo information architecture

**Fig 2:**
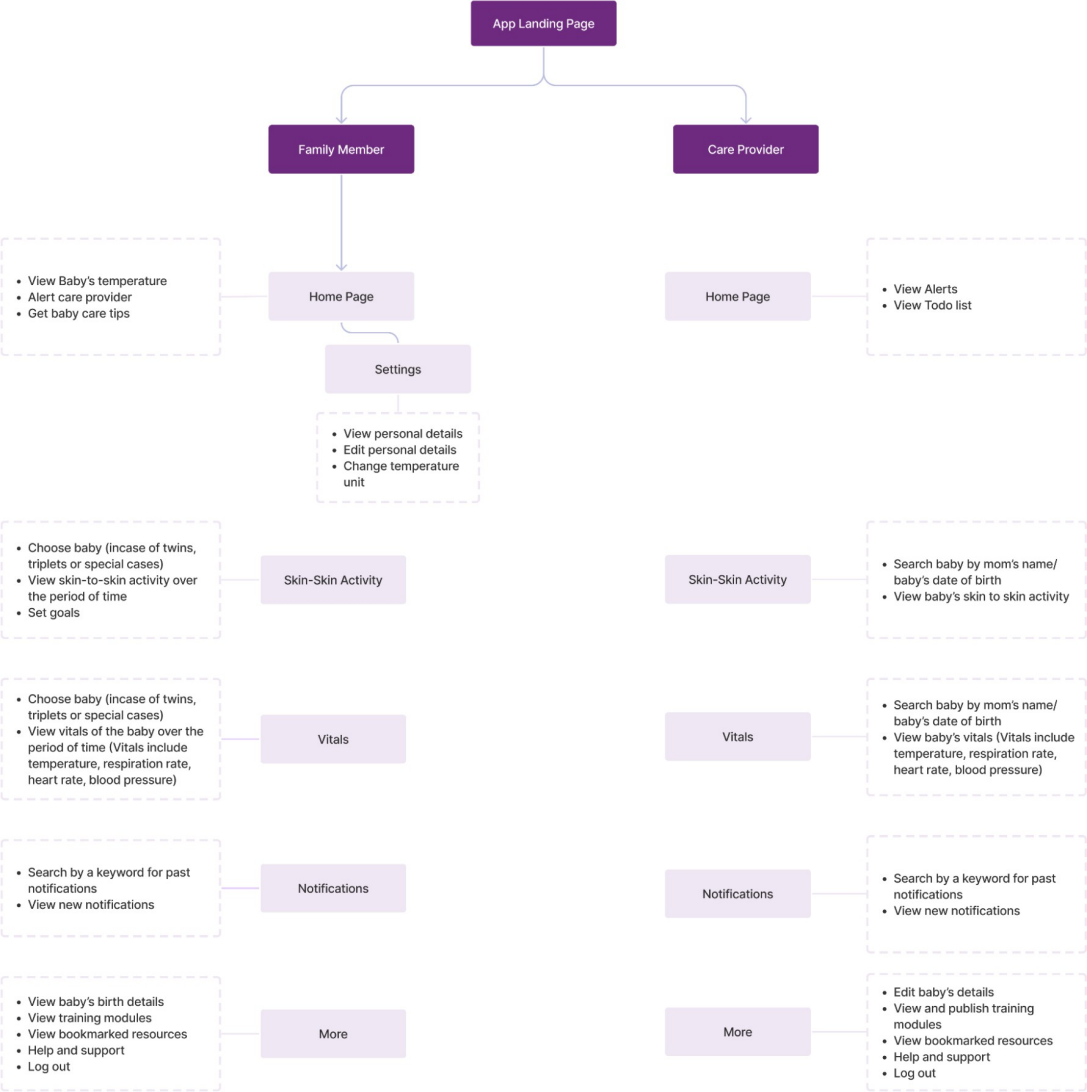
Graphical depiction of NeoRoo information architecture.

After determining initial user requirements, the NeoRoo design and development team engaged in whiteboarding to develop an information architecture schema for the NeoRoo mobile application (Fig 2). The most important desired overall functions of the NeoRoo app are: (a) provide users with essential, up-to-date information regarding the baby’s health and well-being, primarily via real-time vital signs monitoring and automated alerts, and (b) enable the users to utilize the app to act upon this information, as needed. In addition, we also wished for the app to serve as a tool to scaffold targeted information sharing around key evidence-based educational messages for essential newborn care and care of small/premature babies. It was hypothesized that the most effective architecture would be one that allowed for targeted customization of information dissemination to two equally important stakeholders within the premature baby’s ecosystem–parents and healthcare providers.

An important assumption that underpins the design of NeoRoo’s information architecture is that, unlike educational or quality improvement apps, for which “behavior change” might be a desired direct outcome, the NeoRoo approach is that the primary end-users of the apps are already highly motivated and incentivized to engage in activities that maintain and improve the health and well-being of premature babies within their care. Put simply, none of the key stakeholders within a small baby’s environment (parents; healthcare providers; family members; larger communities) wish for the baby to fall ill, or to die. [82] Rather than targeting a need for “behavior change,” then, we hypothesize that the barriers and challenges to the provision of evidence-based newborn care interventions, including KMC/STS within health facilities in LMICs, have less to do with a lack of motivation among families and HCPs to keep babies healthy, and more to do with a paucity of knowledge, empowerment, agency, skills, or access to key tools or resources. The NeoRoo information architecture is built to reflect this hypothesis. Thus, our default position during the whiteboarding process was that adult caregivers of newborns are already highly motivated and incentivized to engage in activities that will keep babies alive and well. The primary purpose of the connected NeoWarm device + NeoRoo app mHealth platform, then, is to collect pertinent information, in real time, about the baby’s health status (e.g., low or high body temperature; apneic episodes), accurately convey this information to parents and healthcare providers in a timely fashion, via color-coded iconography and alerts in the user interface, and utilize the apps’ architecture to equip and empower adult stakeholders to act upon this information, as needed (e.g., parents and healthcare providers can message one another; healthcare providers can utilize the information to ascertain relative acuity of the newborns under their care).

The app is designed with one permission-based access point to achieve this type of information architecture. However, different user interfaces are available depending on what type of stakeholder (parent/family member or HCP) is using the app. In some instances, such as in vital signs monitoring, information is presented to parent/family end-users in a simpler, more straightforward manner. In contrast, the information viewed by HCPs is more detailed, to support clinical decision making. In other instances, the same information is presented identically to both user groups to scaffold shared goal-setting (e.g., skin-to-skin activity module), or to maintain the fidelity of key evidence-based educational and training messages across all stakeholder groups.

### Wireframes

Upon completing the whiteboarding activities and developing the NeoRoo information architecture, the team generated wireframes to visualize an end-to-end workflow for the NeoRoo Family and NeoRoo Healthcare Provider apps. These wireframes served as the basis for the first iteration mock-up of the NeoRoo Family and NeoRoo HCP high-fidelity prototype.

### Iterative prototype development

Figma was utilized to conduct 2 iterations of development for NeoRoo high-fidelity prototypes. During these iterative cycles, we performed internal reviews with subject matter experts and obtained feedback from interaction designers and researchers.

### Iteration 1: Internal reviews with subject matter experts

As previously described, the NeoRoo app design and development team is composed of highly experienced content and subject matter experts. We reviewed the wireframes internally and renamed labels, to ensure clinical accuracy, such as in reporting vital signs monitoring. At this stage, revisions were also made, based on feedback from subject matter experts, to more effectively align the workflow of the apps with the processes and procedures by which daily tasks are typically conducted in a Kenyan KMC/STS ward (NeoRoo Family) and during nursing shifts (NeoRoo Healthcare Provider). A search bar was added to the NeoRoo HCP app, to allow nurses and physicians to search more efficiently through lists of babies under their care. For graphics that demonstrated trends, feedback was received to revise labeling in both the X and Y axes, for improved clarity and accuracy.

### Iteration 2: Feedback from interaction designers and researchers

After seeking functional feedback from internal subject matter experts, we shared the NeoRoo app prototypes with industry-based design professionals and sought their expertise from a visual standpoint. The primary feedback input received from interaction designers during this iterative cycle was in regards to the background color (made it difficult to read the text), the bottom navigation bar (was “clunky” and “heavy,” needed to be streamlined), and depiction of information regarding infant body temperature (placement of the toggle switch for local adaptation by users to the preferred temperature scale of “Celsius” or “Fahrenheit” needed to be shifted).

## Results

Throughout an intensive 7-month iterative design and development phase, we successfully developed a high-fidelity prototype of one permission-based app with two unique user interfaces, each targeted toward specific adult caregivers within a premature/small baby’s care environment. The apps include “NeoRoo Family,” for parents and family stakeholders of small babies in LMICs, and “NeoRoo Healthcare Provider,” for nurses, physicians, and other clinical care staff of premature/small babies.

### NeoRoo Logo

**Fig 3:**
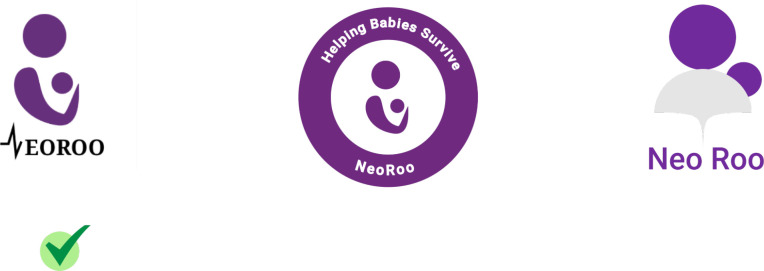
Various iterations of potential logos for the NeoRoo app. The selected logo version is depicted with a green tick.

The NeoRoo app is purposively designed to be feasible and acceptable to any family stakeholder providing care for a premature baby, not solely the mother. Evidence of user requirements analysis emphasized the importance of maintaining gender-neutral colors and iconography, to improve the potential adoption and uptake of the technologies by male stakeholders and the potential for broader acceptance within communities. Thus, in order to remain gender-neutral, no extra cues were added to the NeoRoo logo. Instead, it was constructed with simple geometrical shapes. The “N” within the NeoRoo logo (Fig 3) employs iconography that mimics an image reminiscent of that which might be found on an electrocardiogram trace. This is a nod to the fact that our connected mHealth platform (NeoWarm + NeoRoo) automatically monitors 4 key newborn vital signs, including heart rate. We also hope that, for health care workers, this symbolism will convey that the app will equip them with digital tools to provide evidence-based healthcare for premature babies.

### Shared UI and UX features

In the completed NeoRoo version “A” high-fidelity prototype, many UI/UX design choices, described below, are identical between the NeoRoo HCP and NeoRoo Family apps.

### App landing page and permission-based access

The NeoRoo Version “A” app landing page has screens for permission-based access to user interfaces customized for parents and for healthcare providers. This feature can be customized to require unique passwords or pin codes, as desired by local implementors.

### Iconography

The high-fidelity prototype (“A” version) developed for the NeoRoo app utilizes permission-based access to direct users toward user interface and experience features that are customized for parents/family members and healthcare providers, respectively. As possible, shared iconography is deployed within the UI. Information regarding the continuous monitoring of 4 key neonatal vital signs in premature babies wearing the NeoWarm biomedical device is displayed on the NeoRoo app. The vital signs include body temperature, breathing rate and pauses (e.g., apnea detection), heart rate, and blood oxygen saturation.

### Strategic use of color to depict key information

A number of newborn care educational, training, and clinical care curricula that have been developed by international partners utilize color-coding to emphasize key educational concepts. In particular, the American Academy of Pediatrics *Helping Babies Survive* curricula, and more recently, the interim WHO ENCC courses, creatively employ a “traffic light” color-coding schema to indicate “routine/normal” (green), “caution/warning/needs close monitoring” (yellow) and “danger signs/high risk/emergency” (red). The color-coding schema is a common graphical theme employed across many curricular materials such as facilitator flip charts, action plans, provider guides, and job aids. In developing prior digital health tools within the mHBS/DHIS2 suite, we maintained this color-coding schema across apps. During participatory design feedback and heuristic evaluations of the ECEB/mHBS app, users reported that use of color-coding for particular features and functions facilitated recognition, rather than recall, and reduced cognitive burden, particularly in regards to organizing clinical decision-support information on the user interface. [54,55]

Participants of the user-design sessions for the NeoWarm biomedical device also indicated that color-coded diodes on the strap of the device, which provide information about the baby’s body temperature, are highly desirable. Within the Kenyan setting, there was automatic recognition, among surveyed stakeholders, that a blue LED light indicated “baby too cold,” a green LED light indicated “baby body temperature ok” and a red LED light indicated “baby too hot.” Thus, for NeoRoo, we iterated ways to most effectively augment and display vital signs information and alerts with color-coding. The intent is to provide highly accurate and nearly instantaneous messaging, even to low literacy audiences, regarding the baby’s real-time health status.

**Fig 4:**
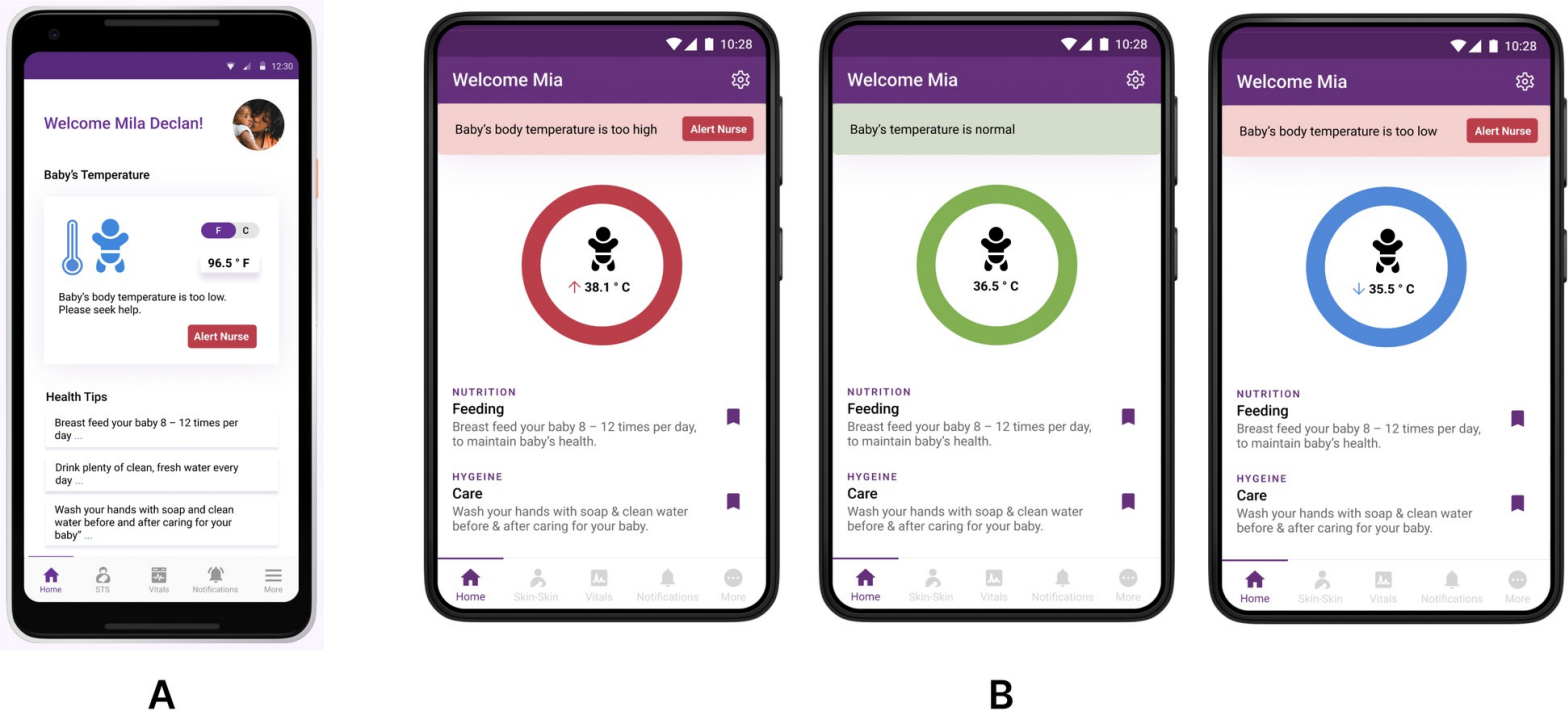
**A-B:** Iterations, across successive cycles of prototype development, of the user interface design for NeoRoo Family home page, which utilizes combined features of color-coding and iconography to depict the real-time body temperature of a simulated premature baby wearing a NeoWarm biomedical device. 4A is iteration 1, and 4B is iteration 2. Please note that all photos and images of persons depicted in the high-fidelity prototype are stock images from Pexels and Flaticon, used under Creative Commons license (additional information and links in Acknowledgements).

Fig 4A and 4B demonstrates successive iterations of the NeoRoo Family app user home page, combining common iconography and strategic use of color. In the first iteration (Fig 4A), color-coded iconography depicting an infant and thermometer indicates the baby is too cold. In a successive iteration (Fig 4B), three types of color-coded rings were displayed, to show the baby’s body temperature status. For infants with body temperature readings that are outside the normal range, the most recent body temperature reading is augmented with color-coded arrows to further indicate if this reading is “too high” or “too low.” The latter iteration also reflects alignment of app UI features with the color-coding schema of the LED diodes on the NeoWarm device itself.

### Vital signs trends over time

An intended function of NeoRoo apps is to provide adult caregivers of premature babies with the ability to see vital signs in real-time, as depicted in the previous section, but also to provide them with digital tools to examine trends in vital signs readings over time. We explored various ways to deploy the color-coding schema to assist in clearly transmitting trend information, even to lower literacy audiences. On the Y (vertical) axis, we experimented with various iterations of a stacked color bar, to demonstrate whether particular body temperature readings are within the normal (green) or abnormal (blue; red) ranges. This feature is reminiscent of a thermometer. It aligns with human-computer interaction principles of the mental model law. The intention was to strategically deploy a common cognitive representation that would, in turn, allow users to have a more intuitive understanding of the information visualized within the graph.[83] The trend line is also color-coded, indicating, over time, how often the baby’s body temperature is normal, too hot, or too cold.

Trend line visualizations are augmented by tool tips. When users click on the “i” icon, they are able to view additional information regarding abnormal body temperature fluctuations. This information can be triangulated with data regarding the trends and duration of KMC/STS, which are depicted in similar graphical displays accessed via the “STS” tab in the toolbar.

### Shared goal-setting for duration of KMC/STS

In order to encourage shared goal-setting for duration of KMC/STS, we provide a feature, within both the NeoRoo Family and NeoRoo Healthcare Provider interfaces, where users can set, and edit, KMC/STS goals by number of hours and minutes, per day, week, or month. This objective is then represented as a “goal line” on the STS activity tracking graph. Duration and trends, over time, of both STS and non-STS care are automatically plotted within the app.

### Aligned educational and training resources

Similar to the other Trainer apps within the mHBS/DHIS2 suite of digital health innovations, the NeoRoo Trainer module allows for linkage with digitized educational training resources from important MNCH partners such as WHO, UNICEF, Save the Children, American Academy of Pediatrics, and Ministries of Health. Within the NeoRoo app, we provide an opportunity for parents and HCPs to develop a small, curated video repository on their Android device. The Global Health Media Project (GHMP) provides access to 226 educational and training videos across a wide range of reproductive health and MNCH topics that are freely downloadable and available in 109 languages. [51] Currently, this on-line library includes 42 videos regarding newborn care, and 27 videos specific to care of small and prematurely born babies, as well as 18 videos regarding breastfeeding.

The NeoRoo app allows end-users to download, store, and watch off-line, around 3 videos at a time. Limiting the video repository within NeoRoo to only a handful of videos at a time is for three primary reasons. First, to ensure that there is not too much storage space taken up on the Android mobile device as a result of accrued videos, which might lead to slow or poor functioning of the device and downloaded apps. Second, because internet connectivity is required to download videos (although, they all play off-line after being loaded on the device), and videos often require a lot of bandwidth, we wanted to reduce the potential frustration, in areas of low internet connectivity, related to slow or poor video download. Third, by allowing for careful curation of a limited, user specific video library, NeoRoo apps adhere to a “just-in-time” concept for educational messaging. Newborn care recommendations can change rapidly over time, as the baby ages, gains weight, and moves through the health service delivery pipeline, from admission to discharge. Thus, different educational content is appropriate for parents and families at various periods. Utilizing the NeoRoo app, HCPs, social workers, or peer mentors can suggest videos and other resources that are the most pertinent resources for each dyad.

In addition to supporting the curation of educational resources related to newborn care and KMC/STS from key partners, the NeoRoo trainer module will also contain a pre-loaded short demo and instructional videos specific to the NeoWarm device. These will be regarding instructional or safety topics such as donning/doffing, safe use, cleaning and disinfection, and troubleshooting technical challenges encountered during use.

### NeoRoo Family vs. NeoRoo Healthcare Provider app customizations

#### Home pages

**Fig 5:**
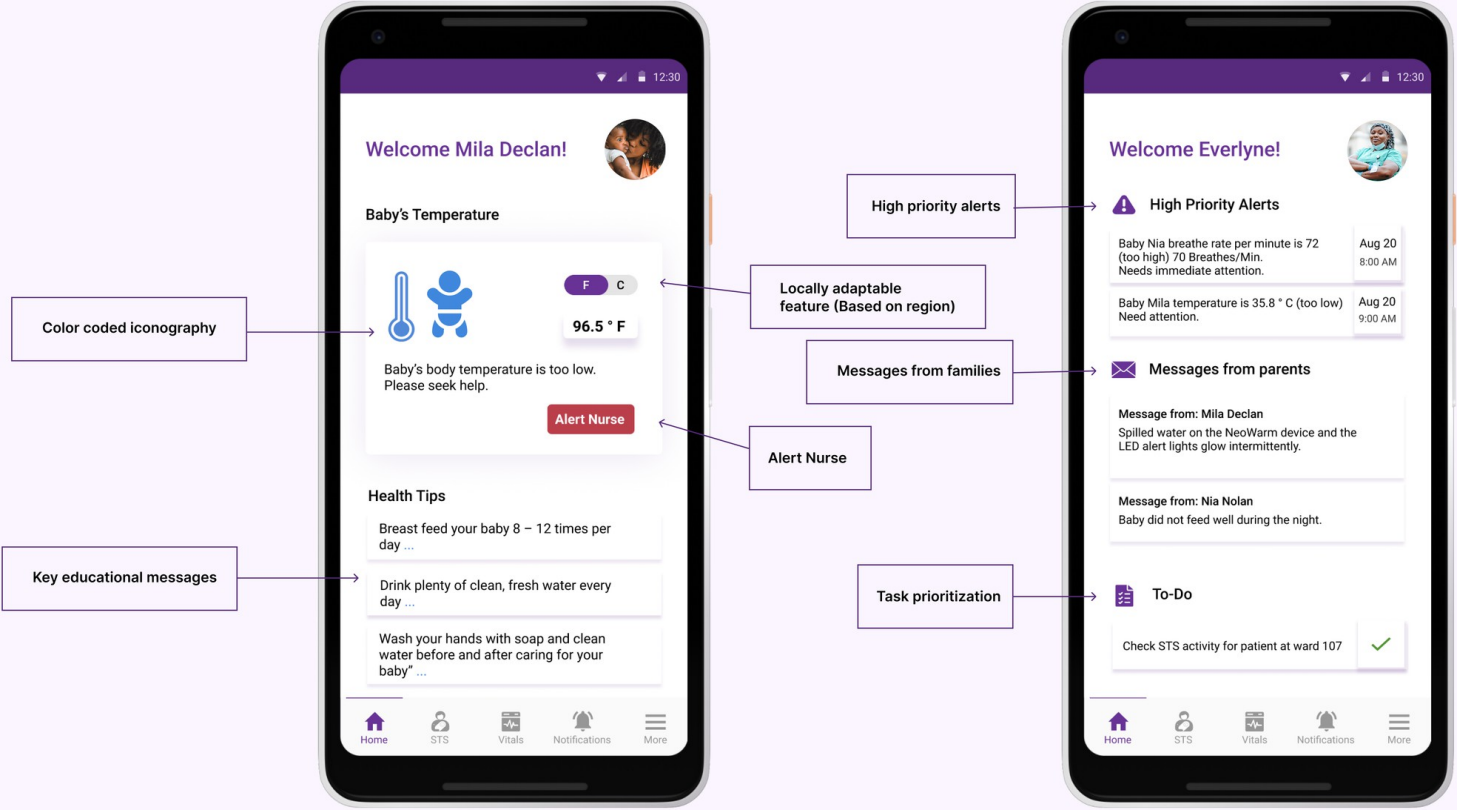
Home pages for NeoRoo Family (left) and NeoRoo Healthcare Provider (right) apps. Key features of the respectively targeted UI designs are highlighted.

The respective home pages of the NeoRoo Family and NeoRoo HCP apps (Fig 5) have been designed to present pertinent information to different adult stakeholders in a targeted fashion. Users of NeoRoo Family are allowed to view only the information about their particular infant (or infants, in the case of multiple-gestation deliveries). They receive instant visual feedback about the health status of their baby, and have the ability, within the app, to send an alert and message to the nurse’s NeoRoo HCP app if they have any concerns. The NeoRoo home page for healthcare providers provides information about any high-priority alerts and parental messages that might require immediate attention. Healthcare providers can generate task lists on the home page to help organize their workflow.

### Skin-to-Skin activity

**Fig 6:**
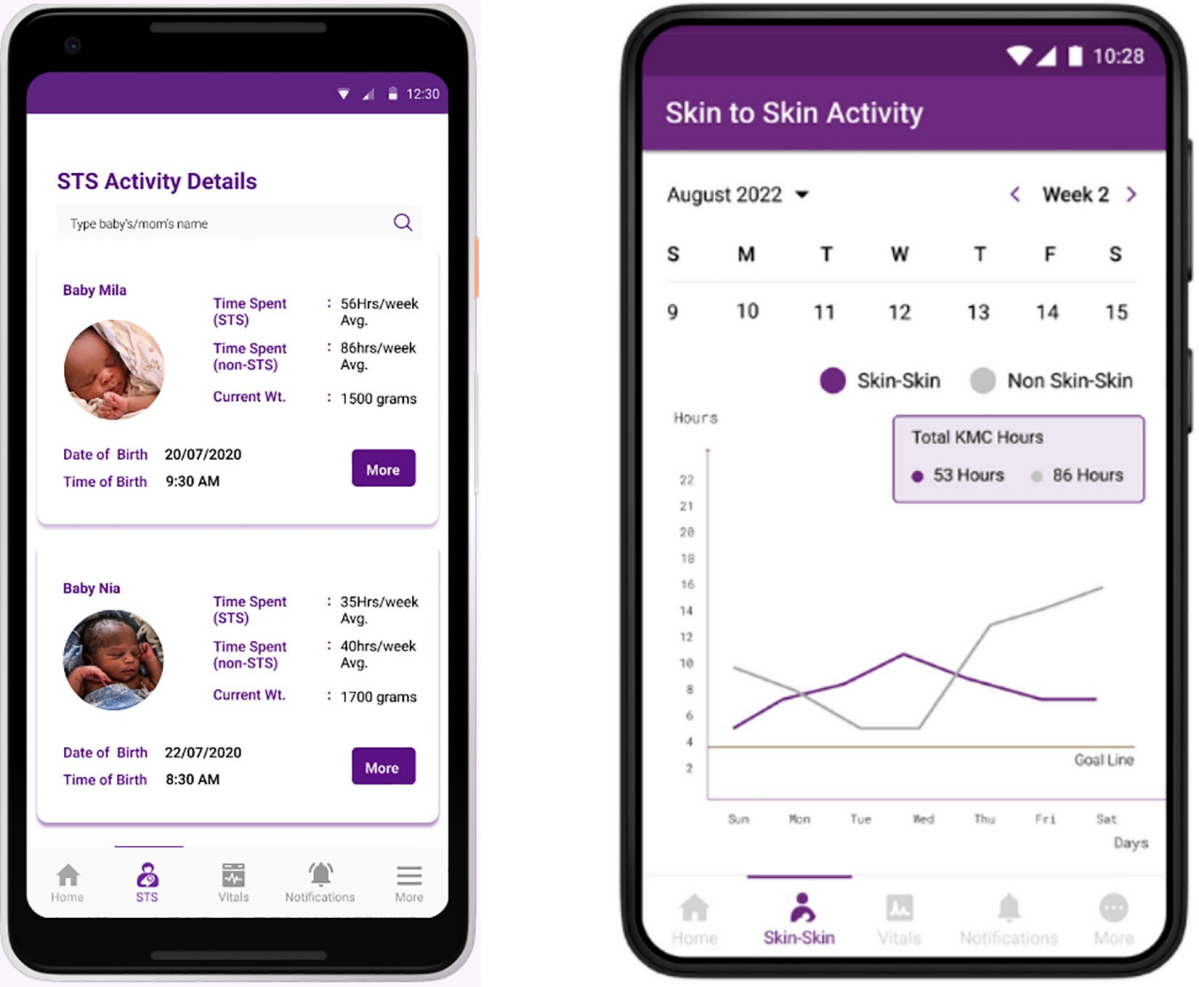
The NeoRoo user interface to display trends in skin-to-skin activity over time and to set daily, weekly, or monthly goals for duration of STS.

Fig 6 shows iterations of the NeoRoo user interface depicting information regarding trends and total duration of hours spent by an adult-baby dyad in KMC/STS. NeoRoo Family app users are only able to view information about their own baby; NeoRoo HCP end-users can view information about all babies under their care. As previously mentioned, both apps have a shared feature in which parents and healthcare providers can collaborate to set collective goals for the number of desired hours of daily, weekly, or monthly hours in which the KMC dyad engages in STS care.

### Functions and features only available to healthcare providers

#### List of babies and access to limited clinical history

**Fig 7:**
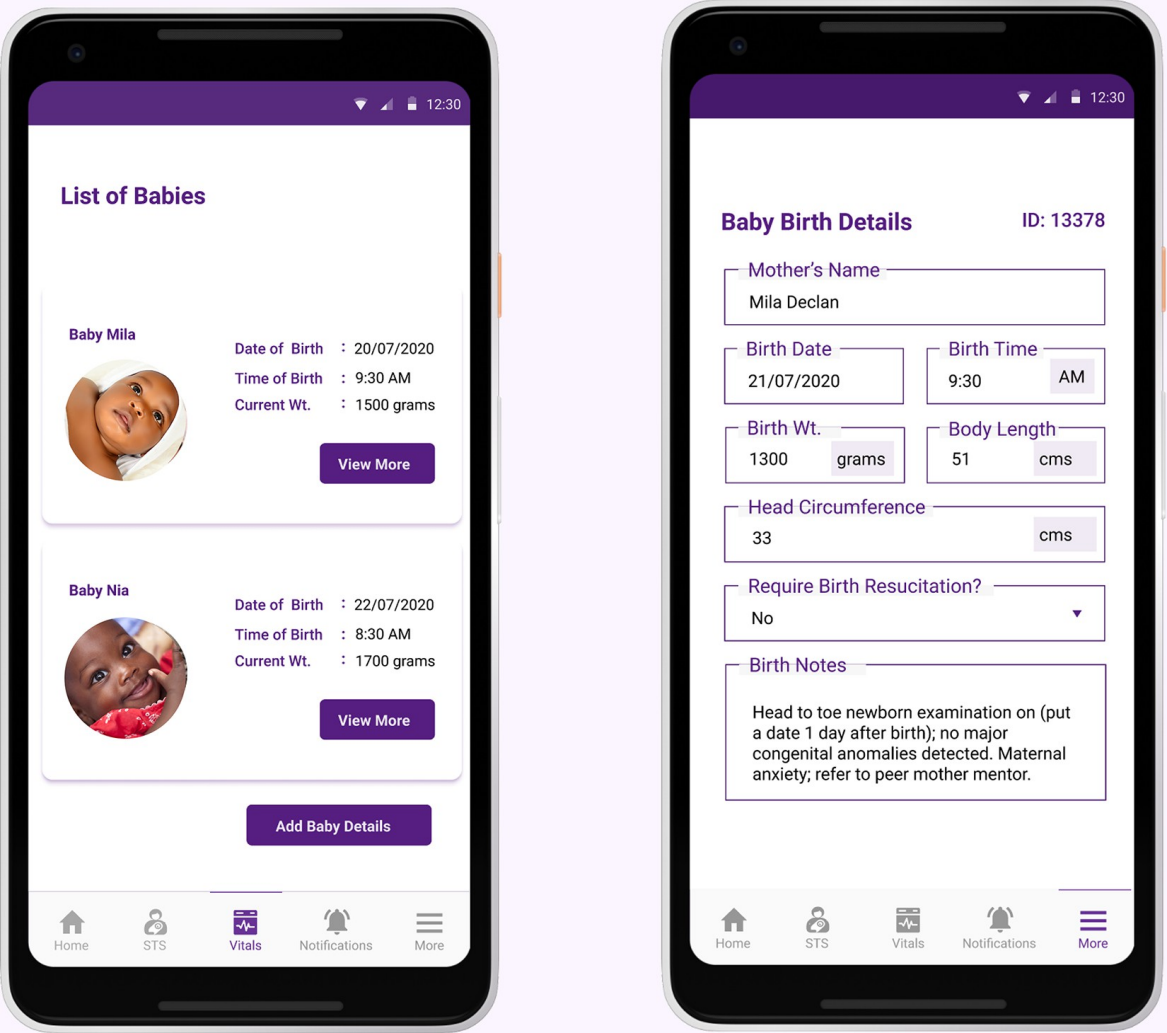
Through the NeoRoo Healthcare Provider app, nurses and physicians can access a list of babies under their care and limited clinical summaries for each infant.

Through the NeoRoo Healthcare Provider app, healthcare providers (nurses; physicians) can access lists of all the babies under their care during a shift. In order to facilitate clinical management, they are also able to access a clinical history summary, such as a very brief description of each baby’s birth history (Fig 7).

## Discussion

Our research initiative aims to develop feasible, acceptable, affordable, and locally-adaptable health innovations that our partners in LMICs can utilize to more effectively implement known, evidence-based interventions to significantly reduce maternal and neonatal morbidity and mortality. We hypothesize that low-cost, open-source digital health innovation (permission-based, customized Android apps for health workers and families, augmented by clinical-decision support algorithms), coupled with a wearable, sensor-enabled, self-warming, swaddling carrier for KMC/STS adult-baby dyads (“NeoWarm”), can be utilized, as part of a thoughtfully integrated mHealth platform, to improve the ability of overburdened adult caregivers in LMICs to care for premature and low birthweight babies more effectively.

In support of this objective, we utilized human/user-centered design techniques and best practices from human-computer interaction to successfully develop a high-fidelity prototype of one permission-based app with two unique user interfaces, each targeted toward specific adult caregivers within a premature/small baby’s care environment.

NeoRoo is being incubated within a global ecosystem that continues to evolve toward increased acceptance and utilization of mHealth and digital health tools. Over the past decade, mobile phone ownership and access to cellular networks have surged. [30–32] In 2022, there were 7.26. billion mobile phone users; 6.64 of these devices are “smart;” with internet access, data storage, and other computing capabilities. This means that over 91% of the global population owns a mobile phone, and 83% own “smart” devices.[84] Acceptance of, and access to, a wide variety of digital health interventions has skyrocketed among a wide range of stakeholders within the mHealth innovation ecosystem, including healthcare providers. [33–37]

The potential advantages of connected mHealth platforms include increased opportunities for more efficient delivery of healthcare services both within, and outside the health facility setting. [32,85,86] The increased penetration of mobile devices among a wide variety of potential end-users and stakeholders, combined with rapid innovation in connected devices, paired with mHealth, eHealth, or telehealth solutions, brings additional opportunities and challenges into this landscape. [87–92] Connected device + app solutions also offer intriguing opportunities for the application of machine learning and artificial intelligence algorithms [93–95] to potentially augment the accuracy and speed of diagnosis, [96] clinical decision -making, [97,98] and secure linkage to portable digital health records via unique user IDs or biometrics. [99]

Challenges include those related to the management of information collected within connected device and diagnostic platforms, and issues around data safety and security, privacy, and management. [88,89,100–103] In addition, an important limitation of our approach is that mobile device access and connectivity still remains out-of-reach for some economically disadvantaged populations, especially in rural settings. To address this particular challenge, the NeoWarm + NeoRoo integrated platform is currently proposed for use within health facility settings, where hospital-purchased hardware bundles (NeoWarm biomedical device + Android smart phones or tablets pre-loaded with the NeoRoo apps) could be provided to KMC/STS dyads during hospitalization and re-used for subsequent patients. Another limitation is that, as described in this paper, the UI/UX choices may not be suitable for low-literacy audiences. We attempted to address this challenge through the development of color-coded iconography, but some features and functions, such as trend graphs, might not be useful for family stakeholders. These are key aspects that we are currently exploring, via participatory design interviews with key stakeholders.

### On-going work and future directions

Currently, our team is performing additional participatory design testing and heuristic evaluation for the “A” version of the NeoRoo app described in this paper. Simultaneously, we have also developed a “B” version of the NeoRoo app. We plan to perform a formal “A/B” split test within Kenyan health facilities in Western Kenya. Eventually, our goal is to conduct a large-scale, global, multisite clinical effectiveness trial, to explore whether, and how, mHealth tools and integrated technologies, such as the NeoWarm biomedical device + NeoRoo mobile apps, might augment the ability of health workers to perform routine monitoring of key vital signs in premature/small babies, and improve compliance, among KMC/STS dyads, to the recommended number of hours skin-to-skin care each day. We seek to understand the manner by which machine learning algorithms, integrated within NeoRoo can assist overburdened healthcare workers in LMICs to more effectively prioritize the healthcare needs of premature babies under their care, and manage workflows in more efficiently. In short, our overarching goal is to develop integrated mHealth solutions that will help to reduce the “know-do” gaps [104] that are often observed when evidence-based interventions, such as KMC/STS, move from the laboratory or tightly controlled research setting, into complex “real world” health ecosystems.

## Conclusions

Utilizing best practices for human-centered, open-source digital health design and mobile app development, our multidisciplinary team used successive iterative cycles to produce high-fidelity mobile app prototypes to equip families and HCPs of premature babies in LMICs to provide evidence-based newborn care, including KMC/STS. On-going participatory design interviews and heuristic usability evaluation will provide key information for additional design iterations.
